# Scheduling of Anaesthesia Operations in Operating Rooms

**DOI:** 10.3390/healthcare9060640

**Published:** 2021-05-28

**Authors:** Pi-Yu Hsu, Shao-Hua Lo, Hsin-Gin Hwang, Bertrand M. T. Lin

**Affiliations:** Institute of Information Management, National Yang Ming Chiao Tung University, Taipei 100-116, Taiwan; k26328172@gmail.com (S.-H.L.); hghmis@gmail.com (H.-G.H.); bmtlin@mail.nctu.edu.tw (B.M.T.L.)

**Keywords:** anaesthesia scheduling, makespan, integer programming, heuristic algorithm

## Abstract

This paper considers scheduling of surgical operations across multiple operating rooms subject to the limited availability of anaesthetists. The objective is to construct a feasible operations schedule that has the minimum makespan, i.e., the completion time of all operations. We abstract the problem into a theoretical server scheduling problem and formulate it in a mathematical form by proposing an integer programming model. Due to the intractability of its computing time, we circumvent the exact approaches and develop two approximation methods. Then, the steepest descent search is adopted for improving the solutions. Computational study suggests that the proposed methods can produce quality solutions in a few seconds.

## 1. Introduction

Medical services in Taiwan are highly accessible with a high coverage of 99% of National Health Insurance. Hospital operations are also increasingly competitive. Healthcare management aims to streamline technical and administrative processes for improving efficiency, productivity, and quality of the healthcare institutions. Operating theaters are one of the major resources that need to be efficiently managed because operating rooms are a type of high-cost units as well as one of the main sources of revenue [1]. Hospitals perform daily operations of operating theaters under the availability of personnel, space, and equipment. One of the most important issues of such management is seeking effective scheduling for maximizing the utilization rate subject to technical, safety, and quality constraints. Scheduling in operating rooms has been receiving considerable research attention for decades (See for instances: Rahimi and Gandomi [1]; Gur and Eren [2], Pinedo [3]; Jung, Kim, and Kim [4]). There is a wide spectrum of research articles on operating room scheduling problems. In general, such settings are coined as scheduling problems subject to the constraints of various types of resources (Cardoen, Demeulemeester, and Belien [5], Lu, Nakao, Shen, and Zhao [6]). We refer the reader to Rahimi and Gandomi [1] for a state-of-the-art review on this subject.

To improve efficiency, some hospitals adopt lean methods or lean thinking approaches to streamline processes, eliminate unnecessary steps, and enhance operational performance and patients’ satisfaction [7,8,9]. This study focuses on another direction in which the operations management issues are investigated through mathematical models of resource-constrained project management and scheduling. Xiang, Yin, and Lim [10] considered the scheduling problem in three-stage elective surgery cases involving multiple resources. The major feature of their model lies in the heterogeneous resource requirements for different stages, including pre-operative stage, peri-operative stage, and post-operative, of the surgeries to be scheduled. Belkhamsaa, Jarbouib, and Masmoudi [11] similarly considered the three-stage multiple-resource scenarios for comprehensive scheduling of selective patient surgeries. Dios, Molina-Pariente, Fern, ez-ViagasAndrade-Pineda, and Framinan [12] developed a decision support system for a 1400-bed, 40-room hospitals. Constraints including resource capacities, time windows, forbidden rooms, and maximum number of rooms a surgeon can attend in a shift. Short-term decisions, medium-term decisions, and manual modifications are provided. Abedinia, Lia, and Yea [13] focused on the blocking situations caused by the limited availability of downstream resources such as intensive care units and post anaesthesia care units. The resource constraints considered by Wang, Meskens, Duvivier [14] are due to personnel characteristics, like affinity between team members and team compositions. Vali-Siar, Gholami, and Ramezanian [15] considered a more general model that involves multiple resources within multiple (5) stages. The addressed resources include personnel, equipment, and beds (pre-operative holding unit, recovery unit, ward, and intensive care unit). This paper singles out the issue of anaesthesia to highlight the availability of anaesthetists and the feature that an anaesthetist is not necessarily present for the whole session of a surgery. We also note that even the singled-out problem is computationally hard to solve.

As indicated by Gur and Eren [2], operations research or quantitative approaches, either deterministic or stochastic, have been deployed to formulate and tackle various optimization issues in operating rooms. A few recent works are introduced. Lin and Chou [16] proposed a hybrid genetic algorithm for minimizing total operating cost that includes the cost for unused idle time and the overtime cost. The recent work Lin and Li [17] designed another meta-heuristic artificial bee colony for minimizing under-utilization waste. Two heuristics based on the shortest processing time first (SPT) rule and the earliest due date (EDD) rule are also applied for computational comparison. Xiang, Yin, and Lim [10] formulated an integer programming model and proposed an ACO algorithm based upon a two-level graph, in which the outer level describes the precedence relations among the surgeries and the inner level describe the whole resources for covering the surgeries. The proposed ACO algorithm is tested through a simulation platform with a comparison with the first-in-first-out scheduling policy. González, Vellasco, and Figueiredo Abedinia, Lia, and Yea [13] formulated an integer programming model for minimizing the likelihood of blocking at the post-surgery stage. For the three-stage comprehensive scheduling problem, Belkhamsaa, Jarbouib, and Masmoudi [11] designed an iterative local search and a hybrid genetic algorithm, which are appraised through real workday benchmark instances. Computational statistics show that their algorithms attained significant reductions in the makespan and the total idle time. In the real application formulated in Vali-Siar, Gholami, and Ramezanian [15], durations of surgery and recovery are uncertain. To hedge against the uncertainty, they deployed a robust optimization model rather than assuming probabilistic distributions of the durations. A constructive heuristic and a genetic algorithm are developed to provide quality schedules. Ref. [18] proposed quantum-inspired evolutionary algorithm for scheduling selective surgical operations to minimize the time for completing all operations and the number of operations out of term. Gür, Eren and Alakaş [19] used integrated goal programming and constraint programming methods to minimize the cost of operating room units, where the cost is measured in negative or positive deviations from the total available time of each room unit in certain time zones. Comprehensive data sets from a state hospital are tested to appraise the performance of the proposed methods under different scenarios. Wang, Meskens, and Duvivier [14] used real data to compare two widely used approaches, integer programming and constraint programming. They found that the integer programming model outperforms for weighted sum objectives, while the constraint programming model has a better performance for the throughput (makespan) objective function. Wang, Li, Chu, and Tsui [20] decomposed the decisions of operating room management into surgery-room allocation with assistant surgeon assignment and surgery scheduling. The objective function of the first stage include room cost and assistant surgeon cost, and the second stage seeks to minimize personnel waiting time cost and overtime cost. They designed a bound-based algorithm that was test through real data collected from 2706 thoracic surgeries. Roshanaei, Luong, Aleman, and Urbach [21] discusses macro decisions across collaborating hospitals and micro decisions within each hospital. They formulated the decision issues into nonlinear programs and deployed various reformulation-linearization techniques to form three mathematical models. Zhang, Dridi, and El Moudni [22] also considered the capacity constraints of the downstream intensive care unit. Uncertainty of surgical and recovery durations render a stochastic programming model, which was then transformed into a deterministic one and handled by a column generation approach.

In this paper, we investigate a scheduling problem of anaesthesia operations in operating rooms. At present, anaesthetists are not widely available, especially in small or medium-size hospitals, in some countries. Some anaesthesia operations are performed by accredited anaesthesia nurses. Therefore, we single out the scheduling issues of anaesthesia operations as a first introduction of such limited availability. Decisions of server scheduling have three folds: assigning jobs to machines, sequencing jobs on each machines, and determining the starting time of each job on each machine. The model considered in this paper involves the last two, assuming that job-machine (operation-room) assignment is settled a priori. The assumption is made from the base that it is not uncommon to have an operation-room assignment in advance due to eligibility issues, especially when special equipment and tools are required. We align the mathematical model of server scheduling and the anaesthesia operations scheduling problem such that the application scenario has its theoretical ground and the mathematical model is associated with more realistic applications. To the best of our knowledge, there are models and algorithms proposed for handling all three decisions simultaneously, or only the last timing decision.

The rest of this paper is organized as follows. Section 2 presents the formal statements of the studied problem in the context of machine scheduling. In Section 3, we formulate the problem into an integer programming model. Due to the computational intractability, we develop two heuristic methods for produce approximate schedules in Section 4. Then, a steepest descent search is adopted for improving the solutions. Computational study is presented in Section 5 to appraise the performances of the proposed resolution approaches. We conclude the paper and suggest future research issues in Section 6.

## 2. Problem Statements

For convenience in description, three operating rooms are considered as an example. The problem, model, and solution algorithms can be extended for more rooms or more anaesthetists. The problem is formerly defined as follows. There are three operating rooms, each k∈{1,2,3} of which has a set of $n_{k}$ operations $R_{k}=\left\{1,2,\ldots ,n_{k}\right\}$ assigned to exercise. Each operation *j* consists of two parts, anaesthesia operation and surgical operation. The required time lengths are denoted by $s_{j}$ and $p_{j}$, respectively. Due to limited availability of resource, only a single anaesthetist is available for carrying out all anaesthesia operations across the three rooms. In other words, at any time instant, no two or more anaesthesia operations can be operated simultaneously. All parameters are assumed to be deterministic, integral, and known *a priori*. The problem is to determine a feasible anaesthesia schedule that has a minimum makespan, i.e., all operations are finished in the shortest time. To illustrate the problem definition, we consider the following numerical example.

**Example** **1.** *Each of the three operating rooms has two operations to perform.*

- *Room 1 has two operations $R_{1}=\left\{1,2\right\}$;*
- *Room 2 has two operations $R_{2}=\left\{3,4\right\}$;*
- *Room 3 has two operations $R_{3}=\left\{5,6\right\}$.*

Their processing lengths of anaesthesia operations and surgical operations are shown below:
**Operations****1****2****3****4****5****6**$s_{j}$323122$p_{j}$435244

**Figure 1:**
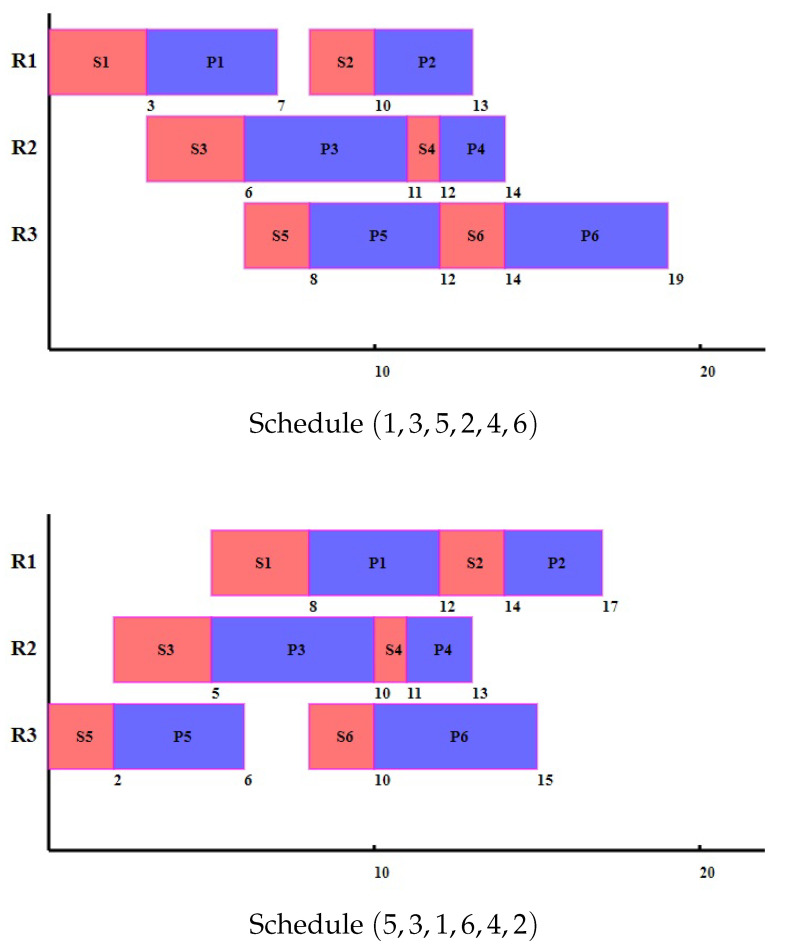
Two example schedules.

We consider two example anaesthesia schedules (1,3,5,2,4,6) and (5,3,1,6,4,2). Their Gantt charts are depicted in Figure 1. Although anaesthesia time $s_{1}$ is longer than those of $s_{3}$ and $s_{5}$ and render two other rooms longer initial waiting times, the final time point in schedule (1,3,5,2,4,6) is only 17, shorter than the makespan 19 of the other schedule (5,3,1,6,4,2).

The abstract model could be considered as a variant of server scheduling on parallel machines, which was first proposed and investigated by Kravchenko and Werner [23]. In the model, two parallel machines are available for processing a set of jobs, each of which consists of two parts, setup and processing. Any machine can process a job at a time, and two two parts of a job should be processed consecutively on the same machine, i.e., interruptions and migrations to another machine is not permitted. All setups can be performed only by a single server, or a skilled technician. In the model, the decisions include dispatching all jobs onto the machines, sequencing the jobs on each machine, and then determining the starting time of each job on each machine. Limited availability of the skilled technician restricts the setups on different machines from being overlapped at any time. A similar scheduling setting is due to the several application contexts addressed in [24,25,26]. They consider scheduling models that consider machines the primary resources and require an assortment of other renewable resources like hoister and skilled worker. Operations or jobs are preemptible, i.e., the jobs are resumable after interruptions. A major difference about operation interruption is that server scheduling requires that setup (anaesthesia) and processing (surgical operation) must be continuous without an inserted idle time.

Werner and Kravchenko [27] showed that to minimize the makespan is NP-hard, even if there are only two machines and all setups take a unit of time. Hasani, Kravchenko and Werner [28] solved the problem by proposing mixed integer programs based on two different block models. Inspired by the application of pilot training program scheduling, Cheng, Krachenkov, and Lin [29] considers the model where preemptions are allowed. Setups and operations can be split into different numbers of pieces with the applications in pilot training. The training courses of a trainee consist of two parts, the first part must be attended with a coach and the second part can be carried out independently by the training. Both parts are divided into 30-min or 60-min sessions. Hong [30] considered scheduling issues in multiple operating rooms with eligibility constraints, i.e., assignment of surgical operations to operating room takes into account equipments sufficiency and technical conditions. Heuristics based on the least-load room first was deployed to produce approximate schedules. Cheng, Kravchenko, and Lin [31] considered a specific model in which the decisions of job assignment and job sequencing are already resolved and given *a priori*. The case with two machines is solvable by a polynomial-time dynamic programming algorithm. It is interesting that the problem becomes strongly NP-hard when there is an arbitrary number of machines. Cheng, Kravchenko, and Lin [32] further showed that the problem remains NP-hard if there are three machines and designed a pseudo-polynomial time algorithm for the case with a constant number of machines. The problem of anaesthesia scheduling considered in this paper reflects the variant in which only job assignment is given to three machines. We need to determine a sequence of jobs and start times of jobs simultaneously on each machine, confining to the non-overlap constraint about setups across all machines. The problem is also intractable to solve.

## 3. Mixed Integer Programming Model

The model is formulated on the decisions that if the setup of a job precedes that of another job. Define binary variables $x_{ij}$ = 1, if job *i* precedes job *j*; 0, otherwise. Denote $t_{j}$ the start time of operation *j*. Then, the completion time of operation *j* is given as $C_{j}=t_{j}+s_{j}+p_{j}$. We want to minimize the makespan

(1) $$\mathrm{MIP}:\mathrm{Minimize}\;\;\;\;C_{\max}$$

subject to a set of constraints as given below.

For any two operations $i,j\in R_{k}$ of the same room, where k∈{1,2,3}, we have

(2) $$x_{ij}+x_{ji}=1,$$

(3) $$t_{j}\geq t_{i}+s_{i}+p_{i}+M\left(x_{ij}-1\right),$$

and

(4) $$t_{i}\geq t_{j}+s_{j}+p_{j}+M\left(x_{ji}-1\right),$$

Constraints (2) enforce the disjoint choices about either *i* precedes *j* or *j* precedes *i*. Constraints (3,4) define the start time of each operation. If operation *i* indeed precedes operation *j*, i.e., $x_{ij}=1$, then $t_{j}\geq t_{i}+s_{i}+p_{i}$ confining that the start time of anaesthesia *j* is no less than the completion time of operation *i*. Also, we have $x_{ji}=0$, which makes Constraints (4) true, nullifying the binding effects of the constraints. The case of $x_{ji}=1$ is discussed in symmetric way.

For operations $i\in R_{k_{1}}$ and $j\in R_{k_{2}}$ with $k_{1}\neq k_{2}$, we note the flexibility for overlapping the anaesthesia operation of a room with the surgical operation of another room. The above set of constraints are adapted as:

(5) $$x_{ij}+x_{ji}=1,$$

(6) $$t_{j}\geq t_{i}+s_{i}+M\left(x_{ij}-1\right),$$

and

(7) $$t_{i}\geq t_{j}+s_{j}+M\left(x_{ji}-1\right),$$

The makespan is confined by the completion of any operation *j* in any room:

(8) $$C_{\max}\geq t_{j}+s_{j}+p_{j},\forall j\in R_{1}\cup R_{2}\cup R_{3};$$

Ranges of decision variables and auxiliary variables are defined:

(9) $$x_{ij}\in \left\{0,1\right\},\forall i\neq j;$$

(10) $$t_{j}\geq 0,\forall j\in R_{1}\cup R_{2}\cup R_{3}.$$

The studied problem is thus defined by Equation (1) through Equation (10) as model **MIP**.

In some application scenarios, the sequence of operations in each room is already determined. We call the model **MIP2**. The problem at the first glance seems to be much simplified. Unfortunately, the decision problem has been proved to be computationally challenging by Cheng et al. (2017). The provisions of fixed sequences are realized in the following constraints.

(11) $$x_{i,j}=1,\forall i<j\in R_{k},k=1,2,3.$$

For $j<j^{\prime }\in R_{k_{1}}$ and $i\in R_{k_{2}}$ with $k_{1}\neq k_{2}$, if $x_{ij}=1$ then we have $x_{ij^{\prime }}=1$. The logic is described as:

(12) $$x_{i,j}\leq x_{ij^{\prime }},\forall j<j^{\prime }\in R_{k_{1}},i\in R_{k_{2}},k_{1}\neq k_{2}.$$

Model **MIP2** is thus defined by Equation (1) through Equation (12).

## 4. Heuristic Algorithms

Since **MIP** and **MIP2** are both computationally intractable. However, the decision time is usually limited. Therefore, in this section, we design heuristic algorithms to produce satisfactory schedules in a timely manner to reflect practical demands.

A basic heuristic is focused on reducing idle times that are resulted in overlapped anaesthesia operations. The first approach is to re-arrange the anaesthesia operations across all rooms in non-decreasing order of their lengths $s_{j}$. Break ties by arranging the operations in non-decreasing order of surgical operation lengths $p_{j}$. Then, each operation is assigned one by one to its room. In other words, the operation with minimum anaesthesia duration has the priority. To describe the heuristics, we define $T_{k,1}$ as the time point of the completion of the last anaesthesia operation on room *k*. Similarly, $T_{k,2}$ is the time point of the completion of the last surgical operation on room *k*. Note that the difference between $T_{k,2}$ and $T_{k,1}$ is the length of the last surgical operation. The steps of our SPT-based heuristic is given in Algorithm 1. An anaesthesia operation can start only if its room is not occupied and the anaesthesia operations of two other rooms are finished. This is prescribed in Line 5.
**Algorithm 1:** SPT-based heuristic
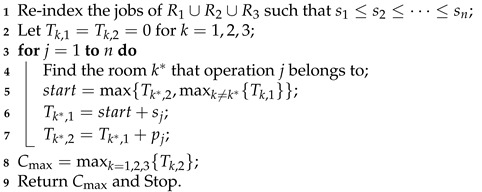


The second approach is to let the least loaded room have the priority to perform its next shortest anaesthesia operation. Algorithm 2 outlines the scheduling steps. To avoid overlapped anaesthesia operations, the newly assigned anaesthesia operation should follow the last anaesthesia operations on two other rooms. This requirement is reflected in $\max_{k\neq k^{*}}\left\{T_{k,1}\right\}$ of Line 7.
**Algorithm 2:** Least-load-based heuristic
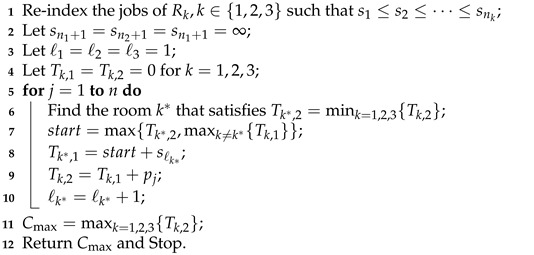


The above two heuristic algorithms assigns operations to rooms from different aspects. Preliminary computational study suggests that the elapsed run times are negligible, meaning that there is room allowing further improvements. Since availability of the anaesthetist is the major concern in scheduling, a plan is intrinsically represented as a permutation of all anaesthesia operations. Therefore, we endeavour to swap operations to see if better schedules are attainable. The steepest descent method is applied, see Algorithm 3. Given a sequence, we enumerate all possible swaps and take the best one, i.e., resulting in the largest reduction in the makespan (Line 9). The procedure iterates the improvement process until no more reduction is possible (Lines 15 and 16).
**Algorithm 3:** Steepest descent improvement
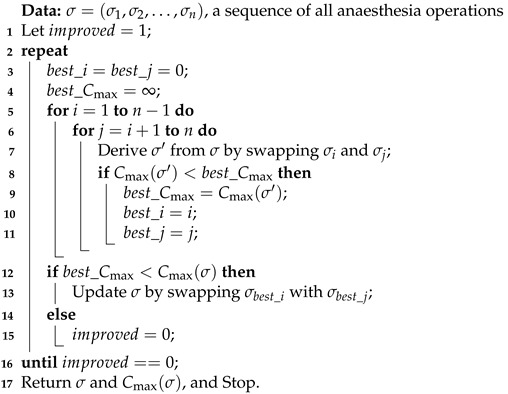


## 5. Computational Study

In this section, we present a computational study to validate the efficiency and effectiveness of the proposed integer programming models and the solution algorithms. The experiments are implemented on a personal computer with an Intel(R) Core(TM) i5-8400 CPU at 2.80 GHz and 8.0 GB RAM. The operating system is Windows 10. All the programs are coded in Python language. The MIP was implemented and solved by off-shell optimization solver Gurobi Version 9.0.3 under a provision license for educational purposes.

In the experiments, all parameters are integer. Lengths of anaesthesia operations ($s_{j}$) were generated from integer uniform distribution [1,10]. Lengths of surgical operations ($p_{j}$) were generated from two different uniform distributions, [1,20] or [1,50] to contrast the relative lengths when compared with anaesthesia operations. The sizes of test instances are determined by the number of operations in each room ($n_{k}$). In the first set of experiments, we consider $n_{k}\in \left\{2,3,5,7\right\}$ and all solution methods will be tested. When the number of operations is larger, the integer program takes a longer time before reaching optimal solutions. The second set of experiments exercise only approximation methods on $n_{k}\in \left\{10,20,30\right\}$, which are large enough for practical situations. For each combination of $n_{k}$ and $p_{j}$, five independent data sets were generated and tested.

In Table 1 and Table 2, the results of all methods are summarized for $p_{j}\in \left[1,20\right]$ and $p_{j}\in \left[1,50\right]$. For the integer program **MIP**, we keep track of the number of instances optimally solve (#opt), the elapsed run time in seconds (time), and the optimal makespan ($C_{\max}$). The cited values of time and $C_{\max}$ are obtained by averaging the corresponding values over the instances optimally solved. Four approximate methods, namely SPT-based heuristic, Least-load-based heuristic, and their counterparts equipped with the steepest descent improvement procedure. The column entitled gap (%) contains the gap between approximate solutions and optimal solutions calculated in percentages as

(13) $$\frac{Z_{A}-Z^{*}}{Z^{A}}\times 100\%,$$

where $Z_{A}$ is the approximate solution and $Z^{*}$ the optimal one. When the run time is less than 0.01 s, it is indicated by a “-”. The statistics indicate that the SPT-based heuristic and the Least-load-based heuristic runs fast. However, the deviations from the optimal values are not satisfactory. Especially, results of the SPT-based heuristic is inferior (more than 30%), although the basic idea is quite intuitive and easy to implement. The phenomenon could be attributed to the idle periods introduced to one room when shorter operations are consecutively assigned to another room. Performance of the Least-load-based heuristic is about 16–18%. It outperforms the SPT-based heuristic because this heuristic gives the least load room the priority to assign an operation so that the idle space in the schedule can be squeezed up to a certain extent. While the two heuristics yield initial schedules, there is room for further improvements. The second phase deploys the steepest descent improvement procedure. The solution quality is significantly strengthened. The gaps drop to around 4–8%. Especially, when the relative lengths between anaesthesia and surgical operations are significant, i.e., $s_{j}\in \left[1,10\right]$ and $p_{j}\in \left[1,50\right]$, the gaps are around 2% except for the outlier case with $n_{k}=7$. We note that the **MIP** solved 3 (respectively, 2) out of the 5 instances with 7 (respectively, 8) operations of each room. In other words, when the problem size increases the exact method cannot find optimal solutions within the specified time limit. An overall appraisal suggests that the proposed two-phase heuristics are effective and efficient when compared with the exact method.

To learn more about the performances of the proposed approximation methods, we tested more instances with 10, 20 and 30 operations assigned to each room. The results are summarized in Table 3 and Table 4. The improvement trend is pretty consistent. The second-phase procedure significantly reduces the scheduling spans. Moreover, the average run time for solving an instance that has 30 operations assigned to each room is less than 4 s. The efficiency renders the decision makers agility in producing quality schedules and the possibility for rescheduling when interruptions and emergency cases present for immediate reactions.

## 6. Conclusions

In this paper, we have formulated scheduling decisions of anaesthesia operations in three operating rooms subject to the limited availability of a single anaesthetist. From a theoretical view point, the problem is computationally challenging to solve. We presented a mixed integer programming (MIP) to prescribe the mathematical notion of the studied problem. The model also allows direct problem solving using off-shell software when the problem scale is small. For instances with more operations, we developed two-phase heuristic algorithms. Computational statistics have evinced the efficiency and effectiveness of the approximation methods, suggesting the potential significance for practical deployment.

For further research, we can extend the model to an arbitrary number of anaesthetists and an arbitrary number of operating rooms. For such more realistic scenarios, the problem is much more complicated since the single-anaesthetist three-room problem is already computationally intractable. To produce a workable, a decent heuristic could be designed by dispatching a free anaesthetist with the lowest recency to the room or operation that is ready for processing. We leave the extension and its algorithm design/implementation for the next stage of research work. Relaxation of the assumption that operations already assigned to operating theaters could be relaxed and made as part of the decisions. Incorporating eligibility constraints concerning facility and personnel renders the problem scenarios more comprehensive models. With new features and relaxations, the problem will become harder to tackle and thus demand efficient and agile solution approaches for attaining better performances.

## Figures and Tables

**Table 1:** Results for $s_{i}\in \left[1,10\right]$ and $p_{i}\in \left[1,20\right]$.

Method	IP			SPT			LL			SPT+SD			LL+SD		
$n_{i}$	**Opt**	**Time**	$C_{\max}$	**Time**	$C_{\max}$	**Gap (%)**	**Time**	$C_{\max}$	**Gap (%)**	**Time**	$C_{\max}$	**Gap (%)**	**Time**	$C_{\max}$	**Gap (%)**
2	5	0.10	40.2	-	52.6	23.57	-	48.4	16.94	0.01	42.8	6.07	-	42.8	6.07
3	5	0.23	57.6	-	83.8	31.26	-	69.6	17.24	0.02	61.4	6.19	-	60.0	4.00
5	5	3.99	90.0	-	136.8	34.21	-	108.6	17.13	0.07	98.0	8.16	0.04	95.0	5.26
7	3	1084.29	123.0	-	197.0	37.56	-	146.2	15.87	0.17	130.8	5.96	0.14	129.4	4.95
8	2	1084.32	137.8	-	218.0	37.08	-	164.8	16.38	0.2	147.4	6.51	0.16	149.0	7.52

-: The run time is less than 0.01 s.

**Table 2:** Results for $s_{i}\in \left[1,10\right]$ and $p_{i}\in \left[1,50\right]$.

Method	IP			SPT			LL			SPT+SD			LL+SD		
$n_{i}$	**Opt**	**Time**	$C_{\max}$	**Time**	$C_{\max}$	**Gap (%)**	**Time**	$C_{\max}$	**Gap (%)**	**Time**	$C_{\max}$	**Gap (%)**	**Time**	$C_{\max}$	**Gap (%)**
2	5	0.23	82.6	-	106.8	22.66	-	91.2	9.43	-	85.8	3.73	0.02	85.0	2.82
3	5	0.31	121.4	-	158.6	23.46	-	130.0	6.62	0.03	123.4	1.62	0.01	122.6	0.98
5	5	4.79	187.6	-	250.8	25.20	-	199.0	5.73	0.09	189.8	1.16	0.07	187.8	0.11
7	5	22.99	234.4	-	353.2	33.64	-	269.4	12.99	0.16	254.6	7.93	0.19	254.8	8.01
8	5	209.57	280.4	-	400.6	30.05	-	299.0	6.22	0.24	282.4	0.71	0.21	284.0	1.27

**Table 3:** Heuristic results for $s_{i}\in \left[1,10\right]$ and $p_{i}\in \left[1,20\right]$.

Method	SPT		LL		SPT+SD			LL+SD		
$n_{i}$	**Time**	$C_{\max}$	**Time**	$C_{\max}$	**Time**	$C_{\max}$	**Imprv. (%)**	**Time**	$C_{\max}$	**Imprv. (%)**
10	-	277.6	-	206.0	0.18	182.8	34.15	0.16	183.6	10.87
20	-	557.0	-	451.0	1.52	377.2	32.28	1.15	378.2	16.14
30	-	991.8	-	671.0	4.44	554.0	44.14	4.75	548.4	18.27

**Table 4:** Heuristic results for $s_{i}\in \left[1,10\right]$ and $p_{i}\in \left[1,50\right]$.

Method	SPT		LL		SPT+SD			LL+SD		
$n_{i}$	**Time**	$C_{\max}$	**Time**	$C_{\max}$	**Time**	$C_{\max}$	**Imprv. (%)**	**Time**	$C_{\max}$	**Imprv. (%)**
10	-	499.8	-	385.2	0.15	346.6	30.65	0.17	348.6	9.50
20	-	968.0	-	872.8	0.87	722.6	25.35	0.87	722.8	17.19
30	-	1494.4	0.01	1209.6	3.57	1065.0	20.73	3.46	1062.4	12.17

## Data Availability

Datasets analyzed in this study can be found at “http://cpanel-199-19.nctu.edu.tw/~bmtlin/healthcare21data.txt” (accessed on 27 May 2021).
